# Supplementary material for: Non plasmonic semiconductor quantum SERS probe as a pathway for in vitro cancer detection
Source: Nat Commun. 2018 Aug 3;9:3065. doi: 10.1038/s41467-018-05237-x (PMC6076273; doi:10.1038/s41467-018-05237-x)
Supplement: Supplementary file 1 — Supplementary Information [file 41467_2018_5237_MOESM1_ESM.pdf]

Supplementary Information

Non Plasmonic Semiconductor Quantum SERS Probe as a Pathway For In Vitro Cancer Detection

Rupa Haldavnekar [a,c], Dr. Krishnan Venkatakrishnan [a,c,d*] Ph.D., Dr. Bo Tan [b] Ph.D.

a. Ultrashort Laser Nanomanufacturing research facility, Department of Mechanical and Industrial Engineering, Ryerson University, 350 Victoria Street, Toronto, ON Canada M5B 2K3

b. Nanocharacterization Laboratory, Department of Aerospace Engineering, Ryerson University, 350 Victoria Street, Toronto, ON Canada M5B 2K3

c. BioNanoInterface Facility, Department of Mechanical and Industrial Engineering, Ryerson University, 350 Victoria Street, Toronto, ON Canada M5B 2K3

d. Affiliated Scientist, Keenan Research Center for Biomedical Science, St. Micheal's Hospital, 30 Bond Street, Toronto, ON Canada M5B 1W8

[*]Corresponding Author: venkat@ryerson.ca 416-979-5000 ext 4984

Supplementary Figures

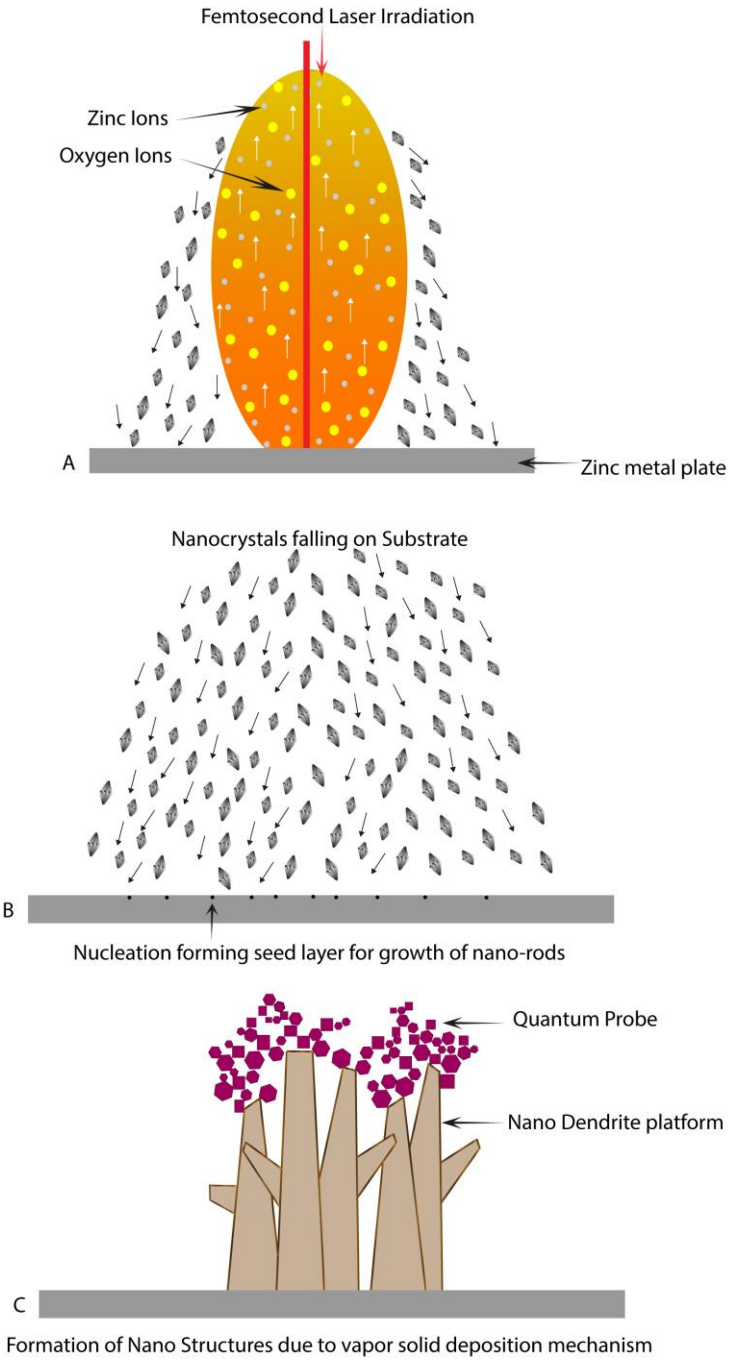

Supplementary Figure 1- Formation of nanostructures due to femtosecond laser interaction.

A: Multi-photon ionization causes formation of ion plume, free electrons in the outer layer of the metal sheet to excite and transfer of energy leads to reconstruction of bonds between zinc and oxygen.

B: When the Zincite/ Zinc nanocrystals fall on the substrate, growth of nanorods due to nucleation takes place. During condensation, the morphology changes into nanowires as well as quantum size crystals.

C: Formation of the nano rods was due to self assembly by melting, collision and coalescence of the nano particles where as formation of 3D quantum probe was due to incomplete coalescence of condensing nuclei.

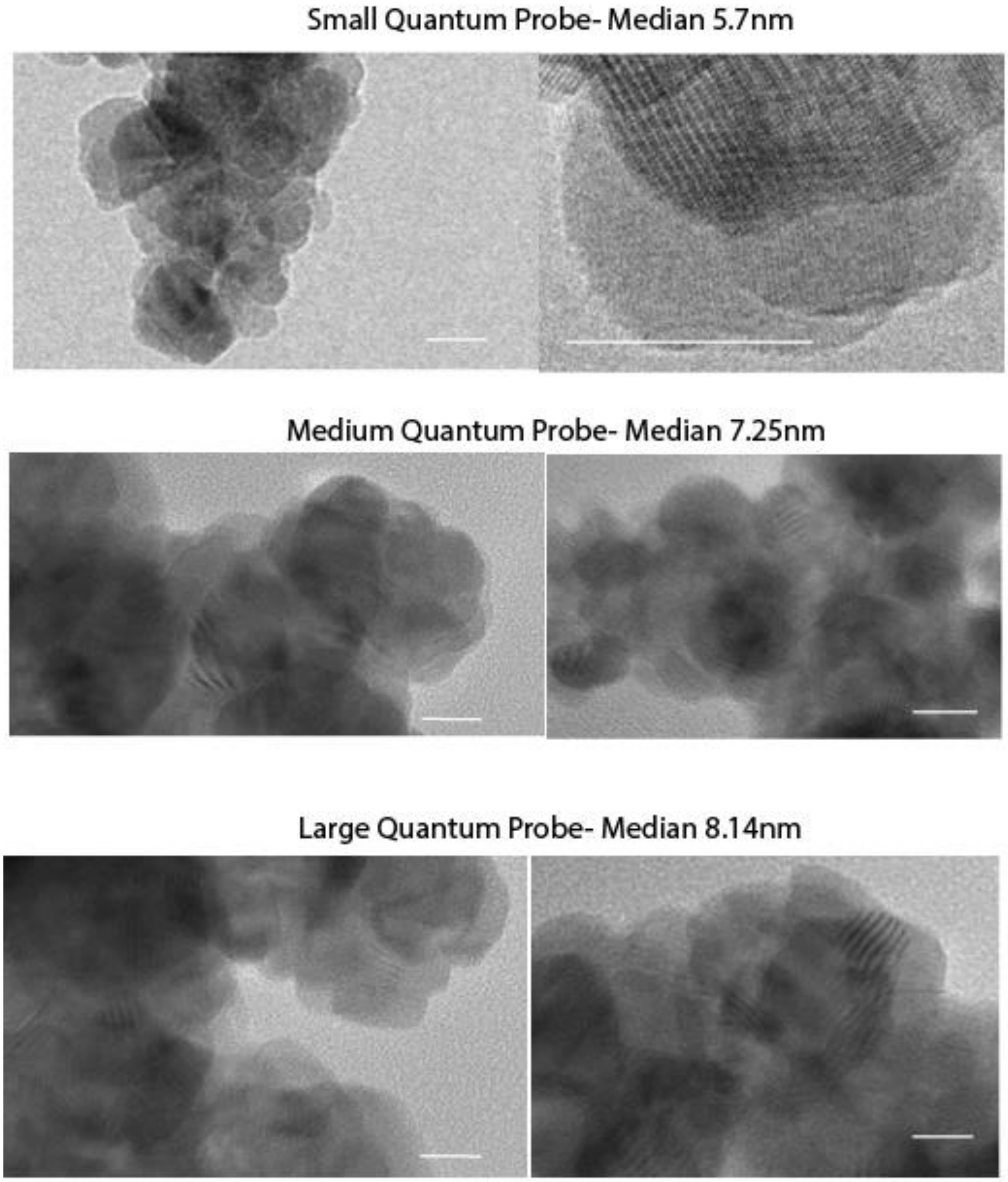

Supplementary Figure 2: Classification of Quantum Probe

Classification of the quantum probe was done based on the size into Small, medium and large. Bar = 10nm

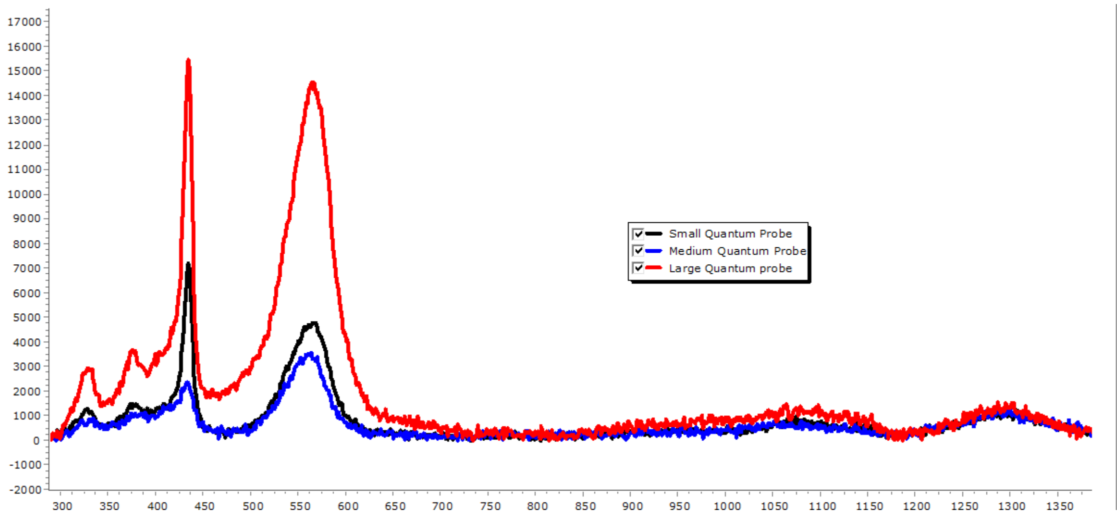

Supplementary Figure 3- Raman spectra of Quantum Probes

Raman spectra of Small, Medium and Large Quantum Probe using 785 nm laser excitation wavelength. The intensity of peak at 438 cm-1 increases rapidly from small to large along-with the sharpness of the peak indicating better crystallinity of the Wourtzite stracture and strong oxygen vibration for the large quantum probe. The “X” axis of the spectra display Raman Shift (cm^{-1}) and “Y” axis displays arbitrary units.

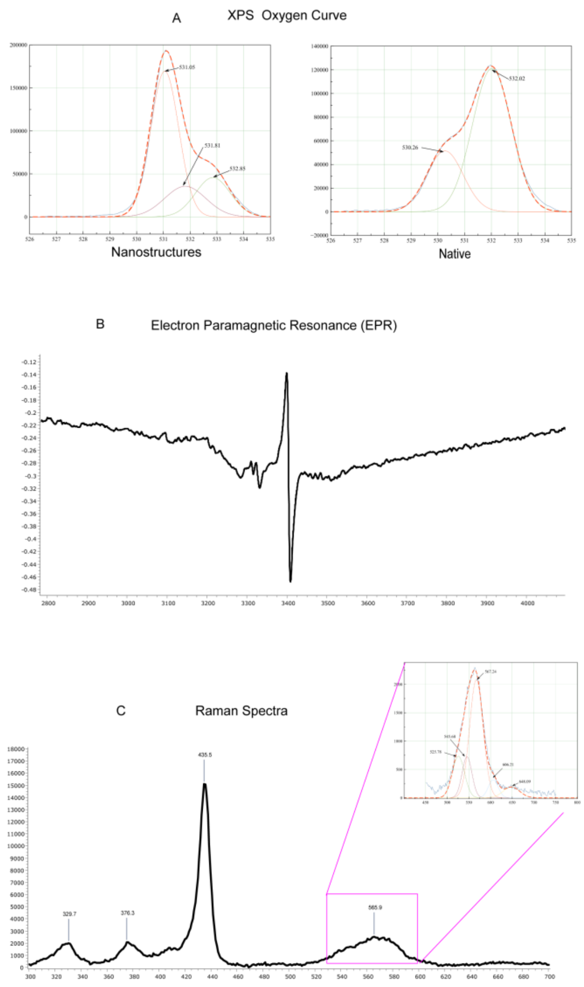

Supplementary Figure 4: Presence of Oxygen Vacancies in the Nanostructures

A: Oxygen XPS spectra revealed changes occurred during native to nanostructure transformation. Deconvulation of the curve demonstrates additional peak 532.85eV assigned to O^- and O^{2-} ion deficient regions. The “X” axis displays binding energy in eV and “Y” axis displays Intensity (Arb. Units)

B: “g factor” from EPR was 1.96 indicating presence of oxygen vacancies. The “X” axis displays field (G) and “Y” axis displays intensity (Arb.Units)

C: Red shift in the phonon modes in Raman spectra also substantiated presence of oxygen vacancies. The “X” axis of the spectra display Raman Shift (cm^{-1}) and “Y” axis displays arbitrary units.

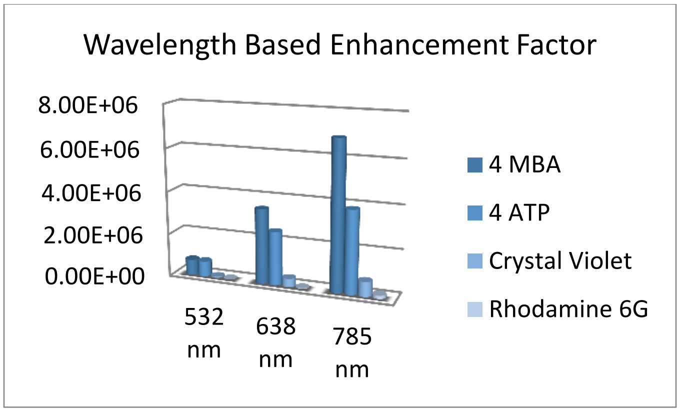

Supplementary Figure 5- SERS demonstrated with ZnO based semiconductor quantum probe

SERS was at multiple excitation wavelengths. The “X”axis displays excitation wavelength (nm) and “Y” axis displays Enhancement Factor

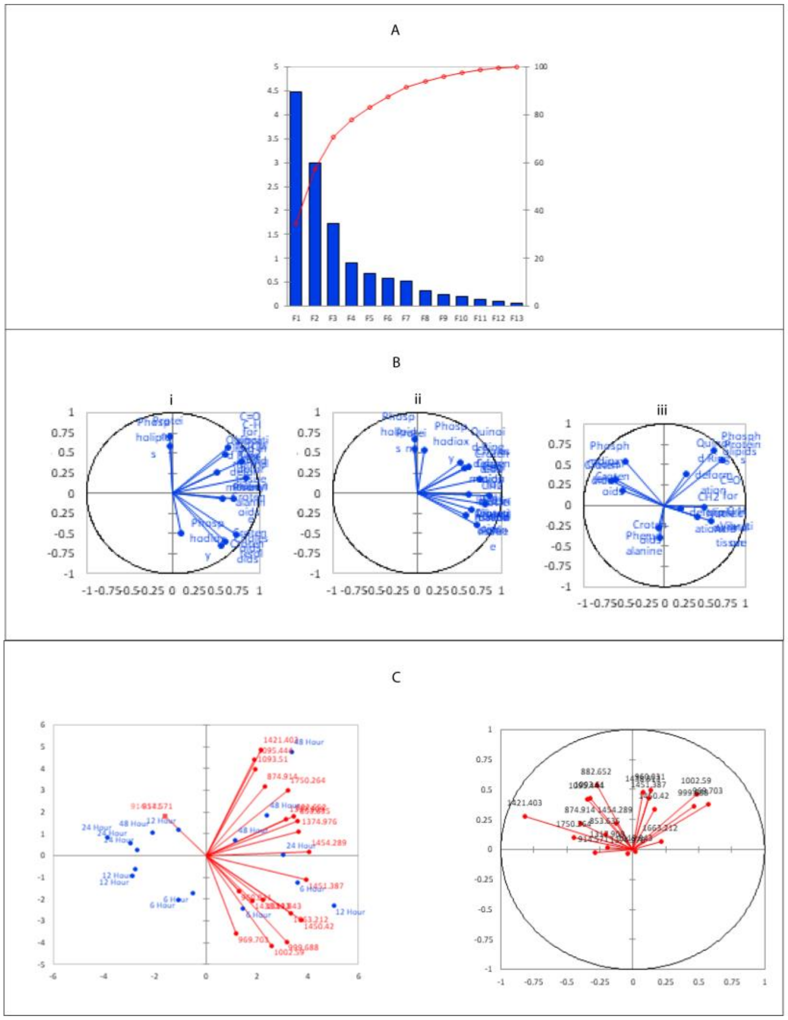

Supplementary Figure 6- Additional Data for Multivariate Analyses

A: Graph of Principal Components for Cell Discrimination. First three PCs cover majority of variance. The “X” axis displays Eigenvectors and “Y” axis displays Eigenvalues (bar chart) & % of cumulative variability (Red line graph).

B: PCs for cell discrimination. (i) Total 57.43% representation , “X” axis displays F1 and “Y” axis displays F2 (ii) Total 47.76% representation, “X” axis displays F1 and “Y” axis displays F3 (iii) Total 36.63% representation “X” axis displays F2 and “Y” axis displays F3.

C: Biplot showing F1 on “X” axis and F2 on “Y” axis for Discriminant Analysis. Variable F1 on “X” axis & F2 on “Y” axis representing 99.88% data.

Supplementary Tables

Supplementary Table 1: Lattice spacing 'd' and planer orientations with Zn-ZnO arrangements observed in the ZnO based semiconductor quantum probe

ZnO Wurtzite Phase		ZnO ZincBlend Phase		Zinc Crystals	
Lattice Spacing A°	Planer Orientation	Lattice Spacing A°	Planer Orientation	Lattice Spacing A°	Planer Orientation
1.7	202	2.66-2.75	002	2.16	101
1.9	220	2.87-3	111	2.39	100
2.45-2.50	101				
2.60-64	002				
2.76-2.83	100				

Supplementary Table 2 : Raman peak assignment for ZnO based Quantum probe and Nano dendrite platform

No	Process	Reference Wave No. (cm^{-1})	Assignment
1	2TA;2E_2^{Low}	203	Multi-phonon process
2	2E_2(M)	329	Multi-phonon process
3	E_2^{High} −E_2^{Low}	333	Second order mode
4	A1(TO)	378	Optical phonon confinement
5	E_2^{High}	438	Oxygen vibration, characteristic wurtzite structure
6	E1(LO)	568	Effects of oxygen vacancies, defects, optical phonon mode
7	2TO	980	Multi-phonon processes
8	TO + LO	1044	Multi-phonon processes
9	A1(TO)+ E1(TO)+ E_2^{Low}	1065	Multi-phonon processes
10	TO + LO	1072	Multi-phonon processes
11	2LO	1105	Multi-phonon processes
12	2A_1(LO), E_1(LO); 2LO	1158	Multi-phonon processes

Supplementary Table 3 – Wavelength based Enhancement Factor Calculation at 1 miliMole concentration

Excitation Wavelength	4 MBA	4 ATP	CV	R6G
532 nm	7.28E+05	7.10E+05	5.50E+04	1.03E+04
638 nm	3.48E+06	2.50E+06	3.90E+05	3.03E+04
785 nm	6.90E+06	3.86E+06	6.99E+05	1.44E+05

Supplementary Table 4 – Molecular b2 band assigenemnts[8] **observed due to SERS from ZnO based non-plasmonic Quantum Probe**

R6G	
613 cm^{-1}	In-plane bending of carbon & hydrogen in xanthenas skeleton
773 cm^{-1}	Out of plane bending of carbon & hydrogen in xanthenas skeleton
1360 cm^{-1}	C-C stretching vibrations mode
1511 cm^{-1}	C-C stretching vibrations mode
Crystal Violet	
1450 cm^{-1}	Ring C-C stretching
1592 cm^{-1}	Ring C-C stretching
4-MBA	
1083 cm^{-1}	Ring breathing mode to the C-S stretching
1146 cm^{-1}	C-H deformation b2
4-ATP	
1078 cm^{-1}	Ring breathing mode to the C-S stretching b2
1167 cm^{-1}	C-H deformation b2

Supplementary Table 5 : Raman Peak Assignments for cellular biomolecules [9], [10]

Wavenumber (cm^{-1})	Peak Assignment
519	v(S-S) gauche-gauche-gauche (amino acid cysteine)
565	Tryptophan/cytosine, guanine
645	C-C twisting mode of phenylalanine- Protein assignment
752	DNA, Symmetric breathing of tryptophan (protein assignment)
820	C-C stretch of proline and hydroxyproline, Out-of-plane ring breathing, tyrosine/O-P-O stretch DNA, Phosphodiester, O-P-O stretching DNA/RNA, Ring breathing tyrosine
915 & 974	RNA Assignment
1030 & 1334	phenylalanine and collagen
1096	DNA, PO_2^- stretching (DNA/RNA)
1126	skeletal of acyl backbone in lipid - trans conformation
1156	C-C, C-N stretching
1209	Tryptophan & phenylalanine n(C-C6H5) mode
1311	bending mode of lipids
1327	CH_3CH_2 wagging mode in purine bases of nucleic acids
1388	CH_3 band
1436	CH2 scissoring vibration - lipid band
1446	G, A (DNA, RNA), CH deformation (DNA/RNA & proteins & lipids & carbohydrates), CH2 bending mode of proteins & lipids
1455	Deoxyribose
1585	C=C olefinic stretch (protein assignment)
1656 & 1660	Lipids
1663	DNA assignment
1684	Amide I (disordered structure; non-hydrogen bonded)
2940	C-H vibrations in lipids

Supplementary Table 6- Eigenvalue of first three dominant PCs (Refer supplementary Figure 6A)

	F1	F2	F3
Eigenvalue	4.137	1.861	1.264
Variability (%)	45.965	20.679	14.042
Cumulative %	45.965	66.643	80.686

Supplementary Table 7- Factor Loading for F1 in PCA

Raman Assignments	Wavenumbers (cm-1)
Deoxyribose	1455
CH2 scissoring vibration- lipid band	1439
A, G (ring breathing of DNA/RNA bases)	1421
Lipid Assignments	1376
G(DNA/RNA) and proteins	1320
G(DNA/RNA) and proteins	1318
DNA	1094
Ribose vibration	974
Ribose vibration	915

Supplementary Methods

Formation of the quantum probe

A pulsed Yb-doped laser was used to synthesize ZnO based semiconductor quantum probes. Some parameters were fixed for maximization of control over the quantum probe and nano dendrite platform. Laser wavelength (1030nm), polarized (circular) and laser power (16W) were fixed to get the optimal nanostructure . The nanostructure were created on 5 X 5mm array of lines with 2 µm point spacing. Samples were in the form of 99% pure zinc plates, surface of which was placed in perpendicular with the propagation of laser beam. The laser processing was carried out by translating the sample plane parallel to sample surface and focus was achieved by translating the sample in perpendicular direction. Scanning speed was varied from 0.5 mm/s to 5 mm/s for control over etch depth and repetition rate was varied from 4MHz to 25 MHz for achieving control peak power. Each ablation area was examined using SEM microscope to confirm the nanostructure formation. Please refer to Supplementary Figure 1, we were able to synthsize ZnO nano dendrite platform decorated with quantum crystals by multi-photon ionization mechanism. The bottom layer is in the form of nano dendrites which are decorated by three dimensional assemblies of quantum probes.

The femtosecond pulsed laser ablation causes rapid fluctuation in the temperature of the plume [1]. Presence of several morphologies of randomly oriented structures like 3-D networks of quantum crystals, random assemblies of nano rods as well as nano wires was observed[2] indicating multiple formation mechanism[3]. Different scanning speeds and repetition rates were used for fabrication. Control on etch depth was achieved by changing scanning speed. By changing repetition rate, control on generation of peak power for each pulse can be achieved. Hence, the energy transmitted to the sample surface can be controlled. As the scanning speed increased, the thickness of the rods decreased and size of the quantum probes increased. As the repetition rate increased, the morphology of the rods changed from thick, blunt and long rods to sharp needlelike long rods to short and thin rods in addition to reduction in size of the crystal mesh. So by programming the synthesis parameters engineering of functionalized the nano dendrite platform for cell adhesion and quantum probe for in-vitro SERS enhancement is possible.

Supplementary Discussion

Raman Spectroscopy Analysis [4]

Raman spectroscopy of all the sample conditions was performed. According to the group theory, each primitive ZnO cell has 4 atoms resulting in 12 phonon branches. Out of these 12 branches, 9 are optical modes and 3 are acoustic modes [3] (Γ_{opt}=1A_1+2B_1+1E_1+2E_2). Polar A_1 & E_1 are infrared and Raman active, E_2 is only Raman active & B_1 is Raman inactive mode. Non-polar E_2 has two wavenumbers E_2(high) and E_2(low). Strong E_2(high) is a characteristic of Wurtzite lattice with good crystallinity. E_2(high) is associated with motion of oxygen and E_2(low) is associated with Zn sub lattice. Going further, the vibration modes of A1 & E1 can polarize the electrostatic field of cells which then splits into longitudinal E_1(LO) and transverse E_1(TO). A sharp peak at E_1(LO) is associated with oxygen vacancies and interstitial Zn.

Refer 'Supplementary Table 2' for peak assignments of ZnO from the literature [2], [5], [3], [6].

Multivariate Analysis of Raman Spectra for discrimination of cell lines

Peaks at 875 cm^{-1}, both cancer cell lines show antisymmetric stretch vibration of choline group N^+ $(CH_3)_3$, characteristic for phospholipids. At 883 cm^{-1}, MDAMB 231 show a very distinct peak for ρ (CH2) protein assignment. At 970 cm^{-1}, MDAMB 231 show overexpression of Phosphate monoester groups of phosphorylated proteins & cellular nucleic acids. This could be due to degenerative stress in the nucleus of cancer cells. Molecular structural signatures can be obtained with Raman spectrum. But, there is an overlap of many peaks in the Raman spectra of relatively large macromolecules like proteins, lipids, Nucleic acids. Thus, many inaccuracies can get introduced in the analysis when the spectra are analyzed qualitatively and certain peaks are assigned to specific biochemical components [11]. These errors are introduced from visual inspection and guessing to determine biochemical component from change in intensity as there always is a possibility of combinational contribution from several components contributing to one peak. Also, there is a possibility of loss of important information from omitted regions of spectra. Thus, chemometric methods of multivariate data analysis were employed for analysis [12].

Twenty Raman spectra from cells located at different locations for each cell line were obtained. There are some prominent peaks observed in the spectra for which peak assignment is done for DNA, RNA, Proteins and Lipids. Main peaks assigned to the biomolecules were shortlisted and the data was compiled for multivariate data analysis. Main information of spectra can be obtained by Principal Component Analysis (PCA). In order to examine qualitative differences and visualize useful information from spectral data, first PCA analysis was attempted. Refer 'Supplementary Figure 6 , Supplementary Table 6 & 7'. F1 interprets 45.96 % of variances, F2 interprets 20.67 % of variances and F3 interprets 14.02 % of variances. Top 3 principal components contributed to 80.68 % of cumulative contribution. This means 80.68 % of information from original spectra is represented in the three dimensional space. In this case, it covers most of the main information. From the cluster graphs, it is evident that there are structural differences in all three cell lines. We were able to discriminate the cell lines with PCA and DA. In order to classify the cell lines, ratio analysis was undertaken.

Supplementary References:

[1] Powell Jeffery Alexander, Tan Bo, and Venkatakrishnan Krishnan, "Programmable SERS active substrates for chemical and biosensing applications using amorphous/ crystalline hybrid silicon nanomaterial," *Sci. Rep.*, 2015.

[2] A. G. Milekhin *et al.*, "Surface enhanced Raman scattering of light by ZnO nanostructures," *J. Exp. Theor. Phys.*, vol. 113, no. 6, pp. 983–991, 2011.

[3] S. J. Chen, Y. C. Liu, Y. M. Lu, J. Y. Zhang, D. Z. Shen, and X. W. Fan, "Photoluminescence and Raman behaviors of ZnO nanostructures with different morphologies," *J. Cryst. Growth*, vol. 289, no. 1, pp. 55–58, 2006.

[4] P. G. Etchegoin, "Quantifying SERS enhancements," *M RS BULLETIN*, vol. 38, pp. 631–640, 2013.

[5] M. Šćepanović, M. Grujić-Brojčin, K. Vojisavljević, S. Bernik, and T. Srećković, "Raman study of structural disorder in ZnO nanopowders," *J. Raman Spectrosc.*, vol. 41, no. 9, pp. 914–921, 2010.

[6] M. Samuel, J. Koshy, A. Chandran, and K. C. George, "Optical phonon confinement in ZnO nanorods and nanotubes," 2010.

[7] W. A. El-Said, T.-H. Kim, H. Kim, and J.-W. Choi, "Analysis of intracellular state based on controlled 3D nanostructures mediated surface enhanced Raman scattering," *PloS One*, vol. 6, no. 2, p. e15836, 2011.

[8] Y. H. Ong, M. Lim, and Q. Liu, "Comparison of principal component analysis and biochemical component analysis in Raman spectroscopy for the discrimination of apoptosis and necrosis in K562 leukemia cells," *Opt. Express*, vol. 20, no. 20, pp. 22158–22171, 2012.

[9] L. G. Quagliano, "Observation of Molecules Adsorbed on III-V Semiconductor Quantum Dots by Surface-Enhanced Raman Scattering," *J. Am. Chem. Soc.*, vol. 126, no. 23, pp. 7393–7398, Jun. 2004.

[10] Z. Movasaghi, S. Rehman, and I. U. Rehman, "Raman spectroscopy of biological tissues," *Appl. Spectrosc. Rev.*, vol. 42, no. 5, pp. 493–541, 2007.

[11] A. C. S. Talari, Z. Movasaghi, S. Rehman, and I. U. Rehman, "Raman spectroscopy of biological tissues," *Appl. Spectrosc. Rev.*, vol. 50, no. 1, pp. 46–111, 2015.
